# A review of 3D In vitro skin models for psoriasis: from pathophysiology to advanced bioengineering

**DOI:** 10.3389/fbioe.2026.1918162

**Published:** 2026-10-05

**Authors:** Yuying Cheng, Feifei Wang, Bo Wang

**Affiliations:** 1 Yunnan Botanee Bio-technology Group Co., Ltd., Yunnan, China; 2 Yunnan Characteristic Plant Extraction Laboratory, Yunnan Characteristic Plant Extraction Laboratory Co., Ltd., Kunming, China; 3 Shanghai Botanee Bio-technology Co., Ltd., Shanghai, China

**Keywords:** 3D bioprinting, 3D skin model, in vitro modeling, organ-on-a-chip, psoriasis

## Abstract

Three-dimensional (3D) skin models have become a transformative choice for replacing animal models in psoriasis research. However, the diversity of platforms, from commercial skin equivalents to organ-on-a-chip systems, has rapidly increased, posing a key selection dilemma for researchers. Based on this, we comprehensively classified the models based on their construction strategies and compared their pathological fidelity from six key dimensions: structural complexity, immune integration, vascular or neural function, scalability, primary application and recommended biological composition. Looking back at the development process of *in vitro* psoriasis models, it can be found that the construction strategy has gradually shifted: from static structural substitutes to dynamic systems that can reproduce natural tissue function. Although commercial models have strong standardization advantages suitable for high-throughput screening, cutting-edge bioengineering platforms including organoids and organ-on-a-chip devices have bridged the gap between laboratory constructs and clinical phenotypes by reconstructing the neural-immune-vascular axis. This review summarizes the latest progress in in vitro psoriasis modeling, explores the bottlenecks that currently exist in the system, and proposes innovative methods to provide new ideas for constructing the next-generation of models that are closer to the pathological characteristics of real psoriasis.

## 1 Introduction

Psoriasis is a chronic inflammatory skin disease with a high recurrence rate, affecting approximately 2%–3% of the global population and showing an increasing prevalence (Greb et al., 2016; Drvar et al., 2019; Griffiths et al., 2021; Yamanaka et al., 2021). Clinically, psoriasis is classified into several subtypes, including plaque psoriasis, pustular psoriasis, erythrodermic psoriasis, psoriatic arthritis, and other variants (Raychaudhuri et al., 2014). Among these, plaque psoriasis is the most common (Le et al., 2019; Benhadou et al., 2020), characterized by well-demarcated erythematous scaly plaques often accompanied by pruritus, which can involve the nails, joints, and even the entire body (Szepietowski and Reich, 2016). In addition to significantly impairing patients’ quality of life (Masson et al., 2020; Garshick et al., 2021; Tang and Chen, 2022), psoriasis is closely associated with multiple systemic comorbidities such as hypertension, diabetes, and cardiovascular diseases, imposing a heavy social and economic burden on affected individuals (van de Kerkhof and van Erp, 2009; Gerdes et al., 2010; Zhang et al., 2015; Long et al., 2021; Pannu and Rosmarin, 2021).

### 1.1 Pathogenesis and clinical burden of psoriasis

Psoriasis is a multifactorial inflammatory disorder that arises from the interplay of genetic and environmental factors (Griffiths et al., 2021; Azuaga et al., 2023), leading to aberrant innate and adaptive immune responses, dysregulated cytokine networks, and excessive autoimmune activation (Kim and Krueger, 2015; Chiricozzi et al., 2018). Despite extensive research, the precise pathogenesis of psoriasis remains incompletely understood.

The pathogenesis of psoriasis involves a complex crosstalk between epidermal keratinocytes, dermal vascular cells, fibroblasts, and immune cells (Kamata and Tada, 2023). As illustrated in our schematic (Figure 1), the process typically initiates with the activation of the innate immune system. Plasmacytoid dendritic cells (pDCs) are activated by complexes of antimicrobial peptides and self-nucleic acids released from damaged keratinocytes (Armstrong and Read, 2020; Tokuyama and Mabuchi, 2020). This triggers pDCs to produce Type I interferons (IFNs) (Nestle et al., 2005), which serve as a critical early signal to activate downstream myeloid dendritic cells (mDCs). Activated mDCs secrete IL-12 and IL-23, polarizing naïve T cells into pathogenic Th1, Th17, and Th22 subsets (Blauvelt and Chiricozzi, 2018; Kim et al., 2018). Additionally, Innate Lymphoid Cells (ILCs) contribute to the inflammatory milieu by rapidly producing IL-17A and IL-22 (De Pasquale et al., 2021). These effector cells release a potent mix of cytokines, primarily IL-17A, IL-22 and TNF-α, which act on various skin cells to drive the disease.

**FIGURE 1:**
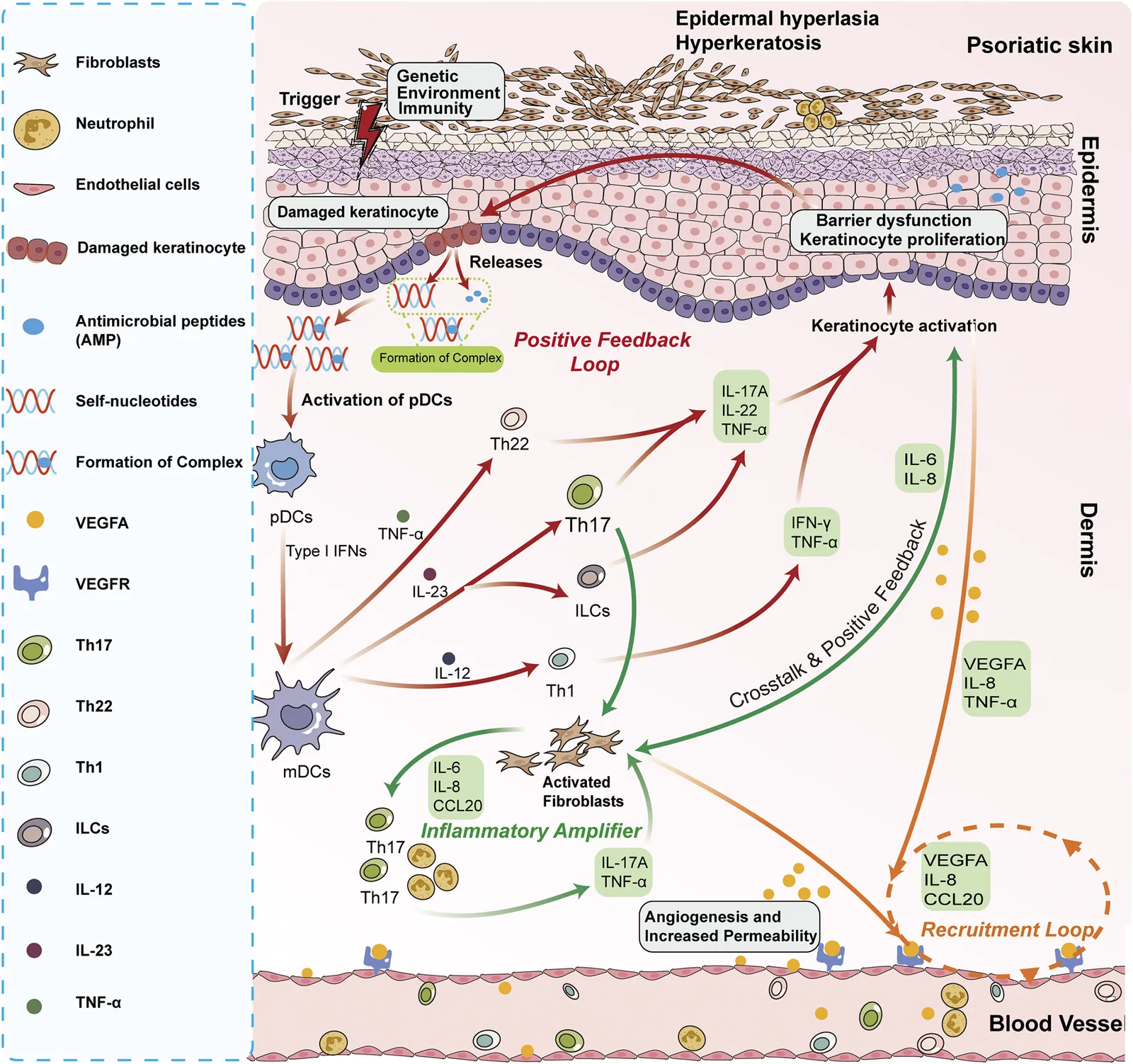
Schematic illustration of the immunopathogenesis of psoriasis. The diagram depicts the complex crosstalk involving immune cells, keratinocytes, fibroblasts, and endothelial cells that drives psoriatic inflammation. Abbreviations: pDCs, plasmacytoid dendritic cell; IFN, interferon; mDCs, myeloid dendritic cell; IL, interleukin; TNF, tumor necrosis factor; Th, T helper; ILCs, innate lymphoid cells; KC, keratinocytes; AMPs, antimicrobial peptides; VEGFA: vascular endothelial growth factor A; VEGFR, vascular endothelial growth factor receptor.

Keratinocytes are the primary targets of these cytokines. Upon stimulation, they proliferate rapidly and differentiate abnormally, leading to epidermal hyperplasia and hyperkeratosis (Yu et al., 2022). They also amplify inflammation by releasing chemokines that recruit more immune cells (Singh et al., 2016). However, keratinocytes do not act alone. Dermal fibroblasts are now recognized as active participants in the inflammatory process (Prens et al., 1990; Debets et al., 1996). When exposed to the psoriatic cytokine milieu, fibroblasts become activated and start secreting various inflammatory mediators, including cytokines (e.g., IL-6) and chemokines (e.g., CCL20, IL-8) (Kamata and Tada, 2022; Ma et al., 2023). This creates a positive feedback loop that amplifies inflammation and establishes a pathogenic dialogue between the dermis and epidermis (Pan et al., 2025).

Meanwhile, the role of endothelial cells in amplifying inflammation is equally critical. Psoriatic lesions are characterized by a dense network of dilated blood vessels and increased angiogenesis (Guérard and Pouliot, 2012). Driven largely by VEGFA and other angiogenic factors derived from keratinocytes and activated fibroblasts, endothelial cells proliferate and increase vascular permeability (Heidenreich et al., 2009). Simultaneously, these activated endothelial cells express adhesion molecules that facilitate the recruitment of circulating immune cells—particularly neutrophils and T cells—from the bloodstream into the inflamed skin tissue (Groves et al., 1991; Terajima et al., 1998; Yazici et al., 2005). Together, these cellular components establish a robust self-sustaining inflammatory circuit that perpetuates chronic inflammation.

In summary, psoriasis is driven by multicellular crosstalk among immune cells, keratinocytes, fibroblasts, and endothelial cells (Elder et al., 2010). Therefore, to truly understand and treat this disease, it is essential to develop *in vitro* models that can accurately replicate this complex cellular ecosystem, rather than focusing on a single cell type in isolation.

Current treatments for psoriasis include topical agents, phototherapy, systemic drugs, and biologic agents (Bachelez, 2017; Ly et al., 2019). Topical therapies, such as corticosteroids and vitamin D analogues, are the first-line option for mild-to-moderate psoriasis (Bhatt et al., 2022; See et al., 2024). They are generally safe and well-tolerated, but their efficacy is limited, and long-term use may cause local side effects such as skin atrophy and irritation. Phototherapy (Group, 1994), particularly narrowband UVB, is effective for moderate-to-severe psoriasis (Yanovsky et al., 2020), especially in patients with widespread lesions who have failed topical treatments (Krenitsky et al., 2020; Sreya et al., 2023). However, it requires frequent clinic visits, carries cumulative dose concerns, and is associated with photoaging and potential phototoxicity with prolonged use. Systemic drugs, including methotrexate, cyclosporine, and acitretin, are reserved for severe or recalcitrant cases (Korman, 2020; van Winden et al., 2020). They are highly effective but have notable safety concerns, including hepatotoxicity, nephrotoxicity, hypertension, and teratogenicity, necessitating regular laboratory monitoring (Kearns et al., 2022). Biologics, such as TNF-α, IL-17, and IL-23 inhibitors, have revolutionized the treatment of moderate-to-severe psoriasis (Gisondi et al., 2020; Bellinato and Girolomoni, 2021). They offer high target specificity, rapid and sustained efficacy, and favorable safety profiles (Hari et al., 2022). However, their high cost, requirement for long-term maintenance, and risk of relapse upon discontinuation remain major challenges, particularly in resource-limited settings (Brownstone ND and Mosca, 2021; Lee and Kim, 2023). Given these limitations, there is an urgent need for reliable preclinical models that recapitulate psoriatic pathology to facilitate drug development and mechanistic studies.

### 1.2 The shift towards 3D In vitro models

Animal models have proven effective for *in vivo* psoriasis research (Schön et al., 2021; Parab and Doshi, 2023). However, species differences and the European Union’s 3R principles (Replacement, Reduction, Refinement) for cosmetic testing have restricted their application in studies of psoriasis pathogenesis and drug development (Asselineau et al., 1986; Jakobsen et al., 2022; Grimm et al., 2023). Given the limitations of animal models and the complex immunopathogenesis described above, the development of advanced *in vitro* psoriasis models has become particularly critical.

Conventional two-dimensional (2D) cell cultures, characterized by monolayer growth, fail to recapitulate the complex cell-extracellular matrix (ECM) interactions and stratified architecture of human skin (Moon et al., 2021; Salminen et al., 2023). These inherent limitations restrict their ability to mimic the dynamic immunopathological features of psoriasis (Nie et al., 2020). Consequently, the development of three-dimensional (3D) *in vitro* models has become a natural progression. By reconstructing the physiological microenvironment and tissue complexity, 3D platforms offer a more biomimetic alternative to both animal models and 2D cultures, enabling a closer approximation to the actual pathological mechanisms of psoriasis.

Unlike previous reviews that focus on specific fabrication techniques, this review adopts a pathophysiology-first perspective, critically evaluating how well each engineering strategy recapitulates the chronic inflammatory loop of psoriasis. Specifically, we first discuss conventional static models that recapitulate early inflammatory responses, followed by advanced bioengineering platforms that incorporate vascular, neural, and immune components to better reflect the complexity of psoriatic pathology. This layered structure allows us to assess the progression of model fidelity alongside the evolving understanding of psoriasis pathogenesis.

## 2 Development and classification of 3D psoriasis models

### 2.1 Conventional 3D skin models

Three-dimensional (3D) skin models, also referred to as human skin equivalents (HSEs) (Idrees et al., 2021) or human epidermal equivalents (HEEs), are artificial skin tissue models constructed via *in vitro* 3D culture technology (Czyz et al., 2023). HEEs consist solely of stratified epidermal structures differentiated from keratinocytes, while HSEs comprise both epidermal and dermal layers, constructed by combining keratinocytes with dermal fibroblasts (Stanton et al., 2022). Fibroblasts rely on natural matrices such as collagen or synthetic scaffolds to form dermal supports (van den Bogaard et al., 2021; Stanton et al., 2022). 3D skin models exhibit reproducibility of human skin in terms of epidermal morphology (Ackermann et al., 2009; Frankart et al., 2012), differentiation, and barrier function. Moreover, they avoid species differences associated with animal models and recapitulate skin stratification and cell-cell interactions, acting as a critical bridge between 2D basic research and clinical translation. Thus, a growing number of researchers are focusing on 3D skin models as *in vitro* platforms for simulating psoriasis.

Currently, commercially available standardized 3D skin models play an indispensable role in in vitro psoriasis research. These standardized platforms offer well-established manufacturing protocols, reproducible experimental results, and low batch-to-batch variability. Representative examples include ready-to-use reconstructed human epidermis (RHE) and full-thickness skin equivalents (FTSE) provided by companies such as MatTek (e.g., EpiDermFT™ (Ampawong et al., 2020)), EPISKIN (e.g., Episkin™ (Scuderi et al., 2022)), and Henkel (e.g., Phenion® FT (Rohrbeck et al., 2024)). To model psoriasis, researchers have developed various induction strategies using these platforms, ranging from direct cytokine stimulation to more complex co-cultures with immune cells and patient-derived cells—aiming to recapitulate the inflammatory loop central to the disease.

#### 2.1.1 Key markers for psoriasis model assessment

Before evaluating these strategies, however, it is necessary to define the criteria by which psoriatic phenotypes are assessed. A core set of markers consistently dysregulated in psoriatic skin is summarized in Table 1.

**TABLE 1:** Key markers in 3D psoriasis models.

Marker type	Core markers	Expression change in psoriasis models	Core functional significance	Representative references
Differentiation Markers	Normal differentiation: FLG, LOR, K10, IVL	downregulated	Maintain skin barrier integrity and reflect abnormal epidermal differentiation	(Boniface et al., 2005; Jean et al., 2009; Smits et al., 2017)
	Abnormal differentiation: K16, K17, elafin (PI3)	upregulated	Mark abnormal proliferation and early differentiation disorders of keratinocytes	(Leigh et al., 1995; Tjabringa et al., 2008; van den Bogaard et al., 2014)
Inflammatory Markers	Core pro-inflammatory cytokines: IL-6, IL-8, IL-17A, TNF-α	upregulated	Drive inflammatory cascades and promote immune cell recruitment as well as keratinocyte proliferation	(Desmet et al., 2017; Rioux et al., 2021)
	Inflammation-related proteins: S100A7, S100A8/A9, DEFB4, IL-36γ	upregulated	Participate in innate immune activation and inflammation amplification; some are psoriasis-specific markers	(Sa et al., 2007; Rioux et al., 2021; Todorović et al., 2022)
Functional Markers	Proliferation: Ki67	upregulated	Reflect the excessive proliferative activity of keratinocytes	(Desmet et al., 2017; Scheurer et al., 2024)
	Barrier function: CLDN1, DSG1, occludin	downregulated	Mark the disruption of tight junctions in the skin barrier	(Scuderi et al., 2022; Morgner et al., 2023)
Pathway-Associated Markers	p-STAT3, p-NF-κB p65	upregulated	Reflect the activation of core pathways	(Sa et al., 2007; Ma et al., 2017)
Immune Cell Markers	CD3^+^, CD4^+^, CD8^+^ T cell infiltration; IL-17A^+^ cells	Infiltration into epidermis/dermis	Reflect T cell recruitment and immune-epithelial crosstalk in psoriatic inflammation	(Rioux et al., 2021; Morin et al., 2023)

Normal differentiation markers such as FLG, LOR, K10 and IVL are predominantly downregulated (Boniface et al., 2005; Jean et al., 2009; Smits et al., 2017); whereas abnormal differentiation markers (Leigh et al., 1995), inflammatory markers (Sa et al., 2007), and proliferation markers are mostly upregulated (Scheurer et al., 2024); barrier function markers are correspondingly reduced (Scuderi et al., 2022; Morgner et al., 2023). Together, these markers reflect the core pathological characteristics of psoriasis, including epidermal thickening, abnormal differentiation, inflammatory activation, and barrier disruption and serve as validation criteria for assessing psoriatic phenotypes in 3D models.

#### 2.1.2 Cytokine-induced psoriatic skin models

A widely adopted strategy for inducing psoriatic phenotypes in 3D skin equivalents is the exogenous application of pro-inflammatory cytokine cocktails that recapitulate the Th17-driven inflammatory cascade (Scheurer et al., 2024; Bhattacharjee et al., 2026). These cytokine-induced models can be constructed on either commercial platforms or laboratory-developed scaffolds, allowing researchers to balance standardization with physiological relevance. Commercial platforms provide well-established manufacturing protocols, reproducible results, and minimal batch-to-batch variation, making them particularly suitable for high-throughput drug screening. In contrast, laboratory-developed scaffolds, such as fibroblast-derived self-assembled matrices, offer enhanced biocompatibility and a more native extracellular microenvironment, albeit with increased technical complexity and longer fabrication times (Mok et al., 2022; Morgner et al., 2023).

For instance, stimulating commercial skin models with exogenous cytokine cocktails has become a widely used approach for high-throughput screening, effectively recapitulating the inflammatory cascade driven by the IL-23/Th17 axis. Rohrbeck et al. (Rohrbeck et al., 2024) employed a commercial full-thickness skin equivalent (Phenion® FT) and induced a psoriasis-like phenotype using a mixture of IL-17A, IL-22, and TNF-α. This method successfully triggered the expression of inflammatory markers, as evidenced by the upregulation of IL-6 protein expression following cytokine stimulation (Figure 2a), highlighting the model’s utility in drug screening. However, the reliance on xenogeneic or synthetic matrices in some commercial platforms can compromise biocompatibility. To overcome this limitation, Morgner et al. (Morgner et al., 2023) developed a full-thickness skin equivalent (FTSE) model using fibroblast-derived self-assembled extracellular matrix (ECM). Upon induction with a Th1/Th17 cytokine cocktail, this model more faithfully recapitulated key pathological features of psoriasis, including the downregulation of epidermal differentiation markers such as FLG and IVL, as well as the upregulation of the psoriasis-associated marker S100A7 (Figure 2b). Similarly, to facilitate the identification and validation of drug targets, Todorović et al. (Todorović et al., 2022) established a dual-model validation system utilizing both self-constructed and commercial reconstructed human epidermis (RHE) models induced with a cytokine cocktail. They demonstrated the utility of this system in dissecting distinct signaling pathways (NF-κB vs. JAK) involved in disease progression. Extending this approach, Scheurer et al. (Scheurer et al., 2024) performed in-depth histological and functional characterization of cytokine-stimulated 3D skin equivalents using advanced techniques Such as multiphoton microscopy-based 3D reconstruction and highly multiplexed CODEX (co-detection by indexing) phenotyping (Figure 2c). They also successfully integrated TH1 T cells into the model, generating an immunocompetent psoriatic construct that exhibited disturbed epidermal differentiation, altered barrier gene expression, and closely resembled psoriatic tissue architecture—providing a robust platform for testing novel treatments.

**FIGURE 2:**
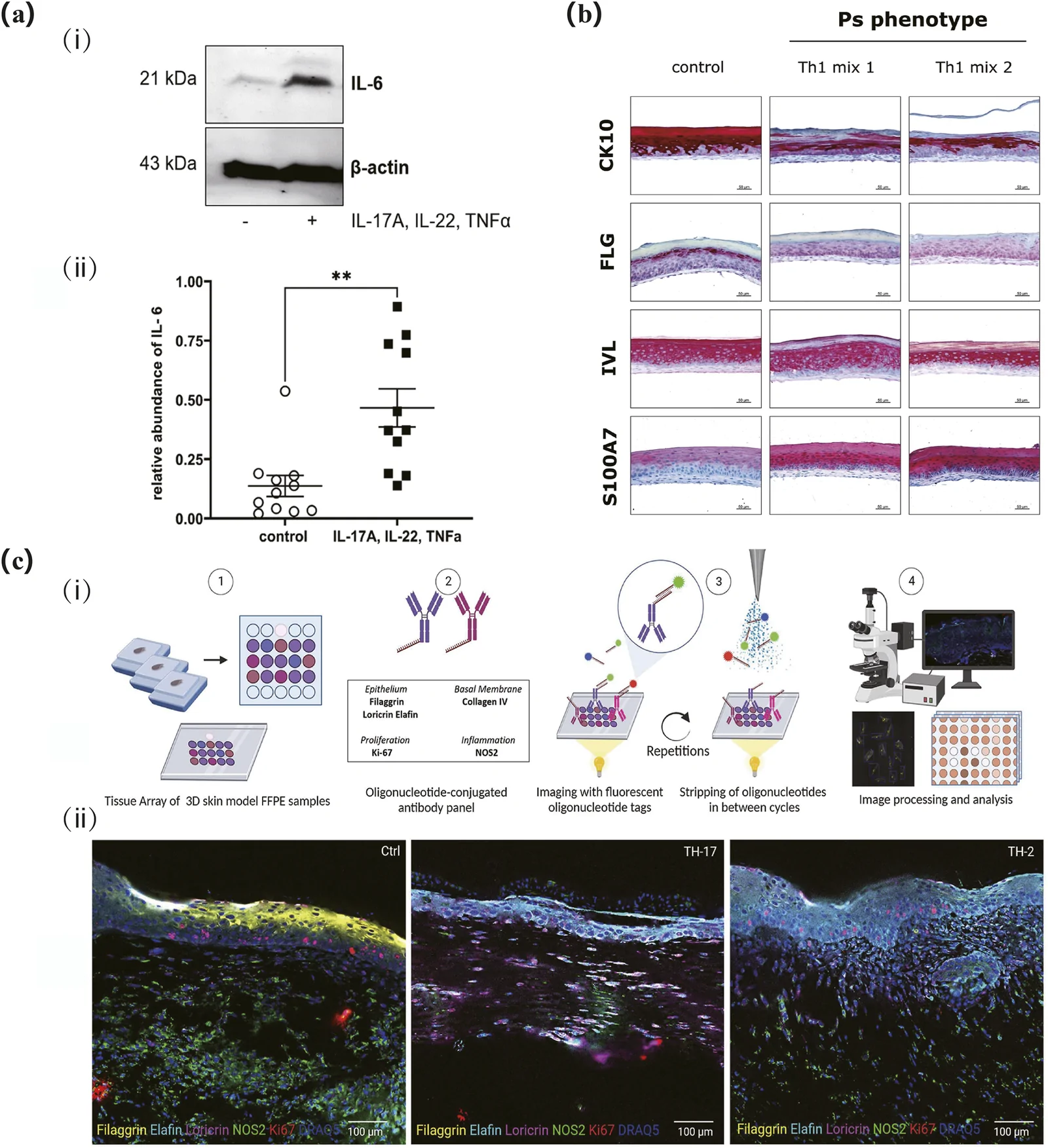
**(a)** Cytokine-induced psoriasis-like phenotype in a commercial full-thickness skin model. (i) Western blot analysis showing upregulation of IL-6 protein expression in the Phenion® FT skin model following 5-day stimulation with IL-17A, IL-22, and TNF-α (10, 25, and 10 ng/mL, respectively). β-actin served as loading control. (ii) Densitometric quantification of IL-6 abundance normalized to β-actin. Data are presented as mean ± SD. **p < 0.01 vs. control. Adapted from (Rohrbeck et al., 2024), Copyright 2024 CC BY 4.0. **(b)** Histological characterization of the self-assembled psoriatic skin model. Immunohistochemical staining of untreated control and Th1 cytokine-stimulated (Th1 mix one and Th1 mix 2) skin equivalents for the differentiation markers cytokeratin-10 (CK10), filaggrin (FLG), involucrin (IVL), and the antimicrobial peptide S100A7. Th1 cytokine stimulation induced downregulation of epidermal differentiation markers (CK10, FLG, IVL) and upregulation of S100A7, consistent with psoriatic pathophysiology. Scale bar: 50 μm. Adapted from (Morgner et al., 2023), Copyright 2023 CC BY 4.0. **(c)** CODEX-based multiplexed imaging of psoriatic skin models. (i) Schematic workflow of the highly multiplexed co-detection by indexing (CODEX) imaging platform, showing antibody conjugation, staining, iterative imaging, and data processing. (ii) CODEX overlay images of untreated control and TH17 cytokine-induced psoriatic skin equivalents, demonstrating simultaneous detection of epithelial markers (filaggrin, loricrin, elafin), proliferation marker Ki-67, inflammation marker NOS2, and nuclear stain DRAQ5. The psoriatic model exhibits reduced filaggrin and loricrin expression with increased NOS2 signal. Scale bar: 100 μm. Adapted from (Scheurer et al., 2024), Copyright 2024 CC BY.

Despite the standardization achieved through these cytokine cocktails and dual-model strategies, these systems inherently lack the cellular complexity of the psoriatic microenvironment. By relying solely on exogenous cytokine, they activate keratinocytes directly while bypassing the antigen presentation and immune cell recruitment that initiate inflammation *in vivo*. Consequently, they fail to capture the reciprocal signaling between immune and stromal cells, and thus the self-sustaining inflammatory loops that drive chronicity. Therefore, to bridge the gap between static induction and physiological relevance, it is essential to move beyond soluble factors and reconstruct the active immune microenvironment.

#### 2.1.3 Immune-integrated and patient-derived psoriatic skin models

This section focuses on the application of immune cell integration and patient-derived cells within traditional, static 3D skin equivalents, pioneering studies that established the feasibility of reconstructing the psoriatic inflammatory loop using these biological components. Commercial skin models can simulate the abnormal epidermal structure characteristic of psoriasis, but they fail to recapitulate the underlying immune-epithelial crosstalk that sustains the disease. The emergence of immune-integrated models addresses this gap by incorporating immune cells or multicellular cytokine networks.

Rioux et al. (Rioux et al., 2021) developed the first immunocompetent psoriasis model by integrating PMA/ionomycin-activated T cells into a tissue-engineered human skin equivalent. By co-culturing these activated T cells with the skin construct, they successfully recreated the inflammatory loop characteristic of psoriasis. This model not only exhibited physiological levels of IL-17A and replicated key pathological features, but also demonstrated predictive validity by responding to anti-IL-17A therapy, as evidenced by the restoration of differentiation markers and the downregulation of inflammatory signatures. This work laid the groundwork for subsequent immune-integrated systems.

While immune integration aims to simulate cellular interactions, Simard et al. (Simard et al., 2021) took a different approach by utilizing fibroblasts and keratinocytes derived directly from psoriasis patients to construct full-thickness skin equivalents. This approach, inherently preserved the disease’s genetic background, enabling the model to successfully replicate not only the classic histological features but also the distinct inflammatory lipid mediator profile associated with psoriasis. This approach demonstrates that integrating patient-specific cells is crucial for capturing the genetic heterogeneity of psoriasis, offering a more authentic platform for studying individual pathological variations.

Building on this foundation, Morin et al. (Morin et al., 2023) advanced the complexity of the microenvironment by establishing a tripartite crosstalk between keratinocytes, fibroblasts, and polarized immune cells. Unlike previous models using generic activation, this study integrated specifically polarized Th1 and Th17 subsets to mirror the distinct immunological endotypes of psoriasis. By introducing these pre-polarized cells into a self-assembled skin model, they created a more physiologically relevant microenvironment that recapitulates the specific contributions of Th1/Th17 pathways. Histologically, psoriatic substitutes with polarized T cells (PS^+^T) exhibited epidermal hyperplasia and increased basal keratinocyte proliferation, as evidenced by increased epidermal thickness and increased Ki67-positive basal keratinocytes (Figure 3a). Immunofluorescence staining confirmed the presence of CD3^+^ T cells, demonstrating the ability of polarized T cells to integrate and migrate within the psoriatic skin model tissues (Figure 3b). Quantitative analysis of culture supernatants demonstrated that IL-17A secretion was robustly elevated in T-cell-containing constructs and significantly suppressed by EPA intervention. In contrast, IL-10 levels were higher in the PS^+^T^+^EPA group than in other psoriatic conditions (PS and PS^+^T); however, neither T-cell incorporation nor EPA supplementation significantly modulated IL-10 secretion (Figure 3c). Together, these data underscore the unique value of such models in dissecting the complex, heterogeneous Immunopathology of psoriasis and paves the way for more personalized disease modeling.

**FIGURE 3:**
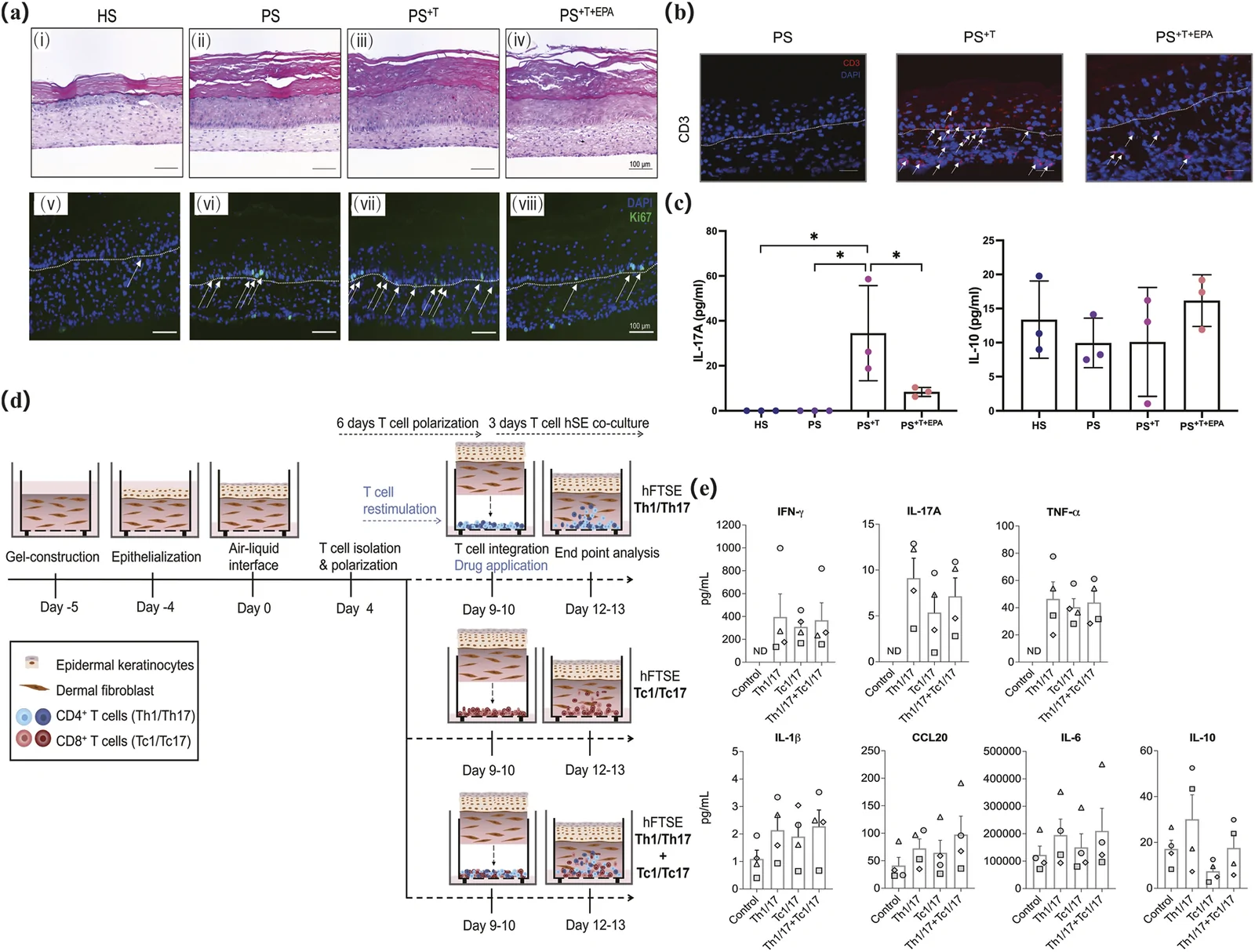
**(a)** H&E staining for histological morphology (i–iv) and immunofluorescence staining of Ki67 for proliferating basal keratinocytes (v–viii) in healthy substitutes (HS), psoriatic substitutes (PS), psoriatic substitutes with polarized T cells (PS^+T^), and psoriatic substitutes with T cells supplemented with EPA (PS^+T+EPA^). **(b)** Immunofluorescence staining of CD3 (T cell marker) in PS, PS^+T^, and PS^+T+EPA^. **(c)** ELISA measurements of IL-17A and IL-10 levels in culture supernatants of HS, PS, PS^+T+EPA^. Adapted from (Morin et al., 2023), Copyright 2023 CC BY. **(d)** Schematic of the experimental workflow. hFTSEs were constructed using fibroblast-populated hydrogels and epithelialization, with 9–10 days of ALI exposure. Parallel *in vitro* polarization of CD4^+^ and CD8^+^ T cells into Th1, Th17, Tc1, and Tc17 subsets was performed over 6 days. These activated T cells were added to the hFTSEs in three combinations (CD4^+^, CD8^+^, or both), followed by 3 days of co-culture and subsequent analysis. **(e)** ELISA measurements of IFN-γ, IL-17A, TNF-α, CCL20, IL-1β, IL-6, and IL-10 levels in culture supernatants of hFTSEs co-cultured with Th1/17, Tc1/17, or combined Th1/17+Tc1/17 T cells, compared to T-cell-free controls. Adapted from (Vahav et al., 2026), Copyright 2026 CC BY.

Extending this approach to encompass CD8^+^ T cells—which are increasingly recognized as key mediators of epidermal inflammation in psoriasis—Vahav et al. (Vahav et al., 2026) recently developed an immunocompetent full-thickness skin equivalent incorporating both CD4^+^ (Th1/Th17) and CD8^+^ (Tc1/Tc17) T cell subsets. The experimental timeline provides a detailed overview of the workflow (Figure 3d). By integrating these polarized T cells into a collagen-fibrin hydrogel-based hFTSE, they demonstrated that both CD4^+^ and CD8^+^ T cells migrate toward the epidermis and secrete psoriasis-associated cytokines. Quantitative cytokine profiling further corroborated these findings, showing that the presence of Th1/Th17 cells potently upregulated the secretion of IFN-γ, IL-17A, TNF-α, CCL20, and IL-1β (Figure 3e). Notably, the presence of CD8^+^ T cells altered the efficacy of the psoriasis drug apremilast, highlighting the importance of including both T cell subsets in preclinical drug testing platforms. Beyond αβ T cells, Huth et al. (Huth et al., 2024) developed an organotypic model incorporating γδ-T cells and demonstrated that the IL-23 inhibitor risankizumab effectively blocked IL-36γ-induced IL-17A upregulation, illustrating how immune-integrated models can elucidate drug mechanisms within complex immune networks.

A critical consideration for immunocompetent skin models is immune compatibility. Most existing immunocompetent models rely on allogeneic co-cultures, where immune cells and skin cells are derived from different donors. This approach carries the risk of triggering non-specific allogeneic immune responses that may obscure the disease-specific immunological pathways of interest. To overcome this limitation, the use of autologous or patient-derived cells is essential for faithfully recapitulating the psoriatic immune microenvironment (Bogomiakova et al., 2023). In this regard, patient-derived induced pluripotent stem cells (iPSCs) offer a promising strategy for generating patient-specific, immunocompatible skin models that preserve the individual’s genetic background, enabling more accurate modeling of personalized pathogenesis and therapeutic responses (Hong et al., 2023). In summary, the integration of immune components has improved the physiological relevance and predictive power of psoriasis models, albeit at the cost of greater technical complexity and lower throughput. Despite successfully recapitulating immune-epithelial interactions, these immunocompetent systems lack a functional vascular network and lymphatic drainage, restricting continuous immune cell recruitment and the simulation of systemic inflammatory responses. Skin-on-a-chip systems address this limitation by introducing perfusable vasculature and dynamic flow conditions to model immune cell recruitment and trans-endothelial migration in a more physiologically relevant context.

Table 2 provides an overview of representative conventional static 3D skin models published between 2020 and 2026. These models illustrate the application of cytokine-induced and immune-integrated strategies within conventional static platforms. Advanced bioengineering platforms, including organoids, skin-on-a-chip, and 3D bioprinting, are discussed separately in Section 2.2, as they introduce additional engineering complexity beyond static culture, such as self-organization, microfluidic perfusion, and precise spatial deposition. While these advanced platforms can also incorporate immune cells and patient-derived cells, their discussion is structured by technological platform rather than by biological strategy to avoid conflating two distinct dimensions of model complexity.

**TABLE 2:** Representative static 3D skin models of psoriasis (2020–2026).

Skin equivalent type	Construction technology	Psoriasis induction	Marker	Ref.
FTSE	Commercial model (MatTek, USA)	Psoriatic keratinocytes	↓IL-10, TGF-β, NRF2, CASP14, IVL, FLG ↑IL-6, IL-8, IL-20, IL-22, TNF-α, CCL-20, S100A7, β-defensin, CASP3	(Ampawong et al., 2020)
FTSE	FN-G nanofilm-coated fibroblasts → 15-layer cell accumulation to form dermis → keratinocyte seeding → ALI culture (day 3)	rhIL-17A (50 ng/mL)	↓FLG, DSG1, CASP14, IGFL2, KRT13 ↑CCL20, CXCL1, CXCL2, CXCL8	(Singh et al., 2020)
FTSE	Self-assembly method: fibroblasts cultured with ascorbic acid for 25 days to form sheets; two sheets superimposed to form dermis → keratinocyte seeding → submerged culture (1 week) → ALI culture (3 weeks)	Patient-derived psoriatic fibroblasts and keratinocytes	↓FLG, LOR, TGM1 ↑K14, Ki67, K17, 12-HETE, LTB4, 9-HODE, PGE2, PGD2, PGF2α	(Simard et al., 2021)
FTSE	Self-assembly method: fibroblasts cultured with ascorbic acid for 28 days to form sheets; activated T cells seeded on one sheet, keratinocytes on another → sheets stacked (T cells in dermis) → ALI culture (3 weeks)	Patient-derived psoriatic keratinocytes and fibroblasts + activated T cells (EasySep isolation + PMA/ionomycin)	↓ Loricrin ↑Ki67, S100A7, elafin, Involucrin, K17	(Rioux et al., 2021)
RHE (Human organotypic 3D skin model)	Ker-CT cells seeded on Transwell inserts → submerged culture (48 h CnT-PR + 24 h CnT-PR-3D) → ALI culture (17 days)	IL-17A (30 ng/mL) + IL-22 (30 ng/mL)	↓FLG, LOR ↑DEFB4, S100A8	(Martínez-Morcillo et al., 2021)
FTSE	Self-assembly method: fibroblasts cultured with ascorbic acid for 4 weeks to form autologous ECM sheets; two sheets stacked → keratinocyte seeding → ALI culture (14 days)	IL-17A (100 ng/mL)	↓KRT10 ↑KRT6, KRT17, hBD2, S100A9, MKi67	(Mok et al., 2022)
RHE	1 Self-constructed RHE, primary human neonatal foreskin keratinocytes cultured at ALI to form 3D RHE 2 RHE from MatTek (Ashland, MA, USA)	Low concentration: IL-17A, IL-22 TNF-α, 1 ng/mL each cytokine High concentration: IL-17A, IL-22 TNF-α 10 ng/mL each cytokine	↓LOR, K10 ↓LOR, KRT10 ↑S100A7,DEFB4, elafin (PI3)	(Todorović et al., 2022)
RHE	SkinEthic™ from EPISKIN Laboratories (Lyon, France, UE)	IL-1A (10 ng/mL) +IL-6 (5 ng/mL) +TNF-α (5 ng/mL) +IL-17A (10 ng/mL)	↓ZO-1, occludin ↑IL-1β, IL-2, IL-12	(Scuderi et al., 2022)
FTSE	Fibroblasts cultured with ascorbic acid for 28 days to form self-secreted ECM sheets → T cells (polarized Th1/Th17) seeded on one sheet, keratinocytes seeded on another sheet → sheets stacked → ALI culture for 3 weeks	Th1: IL-12 (20 ng/mL) + anti-IL-4 (5 μg/mL) Th17: anti-IFN-γ (10 μg/mL) + anti-IL-4 (5 μg/mL) + IL-1β (20 ng/mL) + IL-6 (40 ng/mL)	↓LOR ↑Ki67, S100A7, elafin, IL-17A, IFN-γ	(Morin et al., 2023)
FTSE	Fibroblasts cultured with ascorbic acid for 21 days to form fibroblast-derived matrix → keratinocyte seeding → submerged culture (7 days) → ALI culture (12 days)	Th1 mix 1: IL-17A (10 ng/mL) + IL-6 (10 ng/mL) + IL-22 (25 ng/mL) + IL-1α (10 ng/mL) Th1 mix 2: Th1 mix 1 + TNF-α (10 ng/mL)	↓FLG, CK10, DSG1, CLDN1, LOR, IVL, TJP1 ↑S100A7, CK16, IL-6, IL-8, DEFB4, PI3, LCN2, IL1B, FAS, Ki67	(Morgner et al., 2023)
FTSE	Fibroblasts cultured with ascorbic acid for 21 days to form fibroblast-derived matrix → keratinocyte seeding → submerged culture (7 days) → ALI culture (12 days)	IL-17A (10 ng/mL) + IL-6 (10 ng/mL) + IL-22 (25 ng/mL) + IL-1α (10 ng/mL) +TNF-α (10 ng/mL)	↓FLG, KRT10, CLDN1, TJP1, DSG1 ↑S100A7, Ki67, DEFB4A, PI3, LCN2, IL1B, IL6, IL23A, CXCL1, CXCL8, COX2, IL-6, IL-8	(Morgner et al., 2024)
FTSE	Phenion® FT, Henkel, Germany	IL-17A (10 ng/mL) + IL-22 (25 ng/mL) + TNF-α (10 ng/mL)	↓CLDN1 ↑IL-6	(Rohrbeck et al., 2024)
FTSE	Scaffold-based FTSE with NHEK, NHDF, and γδ-T cells (injected into dermis after epidermal differentiation)	IL-36γ (100 ng/mL)	↑IL-23A, IL-17A	(Huth et al., 2024)
RHE	EpiDerm from MatTek Corporation (Ashland, MA, USA)	TNF-α (50 ng/μL), + IL-17A (50 ng/μL) + IL-22 (50 ng/μL)	↓FLG2, HRNR ↑CCL20, CXCL8, S100A7, IL-36α	(Makino et al., 2024)
Human 3D psoriasis model (commercial)	Commercial 3D psoriatic skin model from MatTek Corporation	Commercial psoriatic model (as purchased)	↓FLG ↑TNF-α, IL-1β, IL-17, IL-22, KRT16, S100A8	(Golob-Schwarzl et al., 2024)
FTSE	1 Fibroblast matrix: fibroblasts seeded 3× over 5 days → cultured 4 weeks → keratinocyte seeding → ALI (7–10 days) 2 Collagen matrix: fibroblasts in collagen I → seeded on collagen-coated inserts → 7 days contraction → keratinocyte seeding → ALI (7–10 days)	1 IL-17A (30 ng/mL) + IL-22 (30 ng/mL) 2 TH1-polarized CD4^+^ T cells (1 × 10^6^) integrated 1 day before ALI	↓FLG, LOR ↑Elafin, Ki67	(Scheurer et al., 2024)
FTSE	Commercial FTSE from MatTek Corporation	IFN-γ (200 ng/mL)	N/A	(Wang et al., 2025)
FTSE	Collagen-fibrin hydrogel populated with fibroblasts → keratinocyte seeding → submerged culture (4 days) → ALI culture (9–10 days); polarized T cells (Th1/17, Tc1/17) added at ALI day 9–10, co-cultured for 3 days	Integration of polarized Th1/Th17 (CD4^+^) and Tc1/Tc17 (CD8^+^) T cell subsets	↓CK10, LOR ↑hBD2, Elafin/SKALP, hBD2, IFN-γ, IL-17A, TNF-α, IL-1β, CCL20, IL-6	(Vahav et al., 2026)
HEEs	Neonatal HEKn cultured at ALI for 28 days	(IL-17 + IL-22, 30 ng/mL each)	↓KRT1, KRT10, FLG, LOR, DSC1, DSG1, CK10, CK14 ↑IVL, DEFB4, PI3	(Bhattacharjee et al., 2026)

Abbreviations: HSE, human skin equivalent; FTSE, full-thickness skin equivalent; RHE, reconstructed human epidermis; RSE, reconstructed skin equivalent; ALI, air-liquid interface; ↑, upregulation; ↓, downregulation.

Marker column lists indicators used to validate successful psoriasis phenotype induction; markers measured after pharmacological intervention are not included in this table.

#### 2.1.4 Application of in vitro models in evaluating clinical biologics

Currently, biologic agents for psoriasis in clinical practice primarily include representative inhibitors targeting the TNF-α(Nicholas et al., 2021), IL-23 (Kauffman et al., 2004; Ghazawi et al., 2021; Daniele et al., 2024), and IL-17 pathways (Hwang et al., 2023). As biologics have evolved from classic TNF-α inhibitors to IL-17 and IL-23 inhibitors with superior efficacy, the application of *in vitro* models has similarly evolved from foundational validation to mechanistic elucidation.

Early studies established the foundation for biologic assessment. For instance, van den Bogaard et al. (van den Bogaard et al., 2014) constructed a skin equivalent model containing activated T cells and demonstrated that the anti-TNF-α drug Infliximab significantly reduced TNF, DEFB4, and CXCL8 expression. As model technologies matured, their application shifted towards evaluating the pathological reversal capacity of novel agents. Singh et al. (Singh et al., 2020) utilized an IL-17A-induced psoriasis model to show that the anti-IL-17A antibody Secukinumab downregulated pro-inflammatory cytokines and chemokines, while restoring differentiation markers such as filaggrin. More recently, Huth et al. (Huth et al., 2024) developed an advanced model incorporating γδ-T cells and revealed that the IL-23 inhibitor Risankizumab blocked the IL-36γ-induced IL-17A upregulation. Collectively, these studies demonstrate that *in vitro* models have evolved from simple platforms for drug screening into precision systems capable of elucidating drug mechanisms within complex immune networks. This progression highlights the growing utility of these models in guiding next-generation therapeutic strategies.

### 2.2 Advanced bioengineering platforms

To overcome the limitations of static culture and avascularization inherent in conventional models, advanced bioengineering platforms have emerged. Unlike the static models discussed in Section 2.1, which rely on exogenous scaffolds and random cell seeding, these advanced platforms introduce distinct engineering dimensions: self-organizing organoids with appendage formation, microfluidic skin-on-a-chip systems with perfusable vasculature, and 3D bioprinted constructs with precisely defined spatial architectures. Importantly, while these advanced platforms can also incorporate immune cells and patient-derived cells, our classification in this section is based on technological platform rather than biological strategy, reflecting the additional engineering complexity they introduce. Therefore, Table 2 focuses exclusively on conventional static models, while the following subsections discuss representative studies of each advanced platform.

Critically, rather than cataloging technological features for their own sake, our analysis anchors each platform in the specific pathological question it enables us to address: how do appendage-resident immune cells contribute to disease chronicity, how do hemodynamic forces regulate vascular-immune crosstalk, and how does spatial tissue heterogeneity influence inflammatory signaling? This pathophysiology-first lens shifts the evaluation from what a technology can build to what a technology can reveal about disease mechanisms.

#### 2.2.1 Psoriasis organoids

Among these advanced platforms, skin organoids represent a significant leap in recapitulating structural complexity. While immune-integrated models have successfully reconstructed the epidermal-dermal interface, they largely lack skin appendages (Zhang et al., 2021; Hosseini et al., 2022), such as hair follicles and sebaceous glands (Takaya et al., 2022), which serve as critical functional microenvironmental components rather than mere structural features. These appendages act as hubs for stem cells and immune cells, playing a pivotal role in the pathogenesis and recurrence of psoriasis. Specifically, hair follicles serve as a reservoir for resident memory T cells (T_RM_) (Adachi et al., 2015), which are key drivers of psoriatic relapse (Ryan et al., 2021; Ghaffarinia et al., 2023).

The presence of these appendages is not merely a structural feature but creates distinct functional microenvironments that influence immune cell retention, activation, and disease chronicity (Dainichi and Iwata, 2023). The absence of such structures in conventional skin models therefore limits their ability to recapitulate the full complexity of psoriatic inflammation and relapse mechanisms (Hosseini et al., 2022). Skin organoids, through their intrinsic self-organization capacity, offer a transformative platform to overcome this anatomical deficit by enabling the formation of hair follicles, sebaceous glands, and other appendages *in vitro* (Sandoval et al., 2023), thereby providing a more physiologically relevant microenvironment for studying the role of appendage-resident immune cells in psoriasis pathogenesis and recurrence.

To address this specific anatomical deficit, skin organoids have emerged as a transformative platform. Skin organoids are defined as three-dimensional, self-organizing microtissues derived from stem cells that recapitulate the architectural and functional complexity of native skin. Unlike traditional skin equivalents that rely on exogenous scaffolds and random cell seeding, organoids possess intrinsic self-organization capacity (Fang et al., 2023), enabling the formation of complex structures including hair follicles and sebaceous glands. Based on their cellular origin, skin organoids can be broadly classified into two categories: (1) pluripotent stem cell (PSC)-derived organoids, generated from embryonic stem cells (ESCs) or induced pluripotent stem cells (iPSCs) through directed differentiation; and (2) adult stem cell (ASC)-derived organoids, established from tissue-resident progenitor cells isolated from skin biopsies. Among these, iPSC-based platforms are particularly promising for disease modeling, as they preserve patient-specific genetic backgrounds while enhancing the generation of complex, appendage-bearing skin structures (Zhao et al., 2011; Guo et al., 2013). Importantly, PSC- and ASC-derived organoids are complementary rather than mutually exclusive: PSC-derived organoids excel at recapitulating developmental processes and complex architecture, while ASC-derived organoids better preserve the epigenetic memory and microenvironmental characteristics of the original tissue.

Although there are currently few reports on psoriasis-specific organoid models, significant progress has been made in normal skin organoids construction, which has pointed the way for psoriasis research. Ahmed and colleagues have made substantial contributions in this area through a series of studies. Their team developed a robust and optimized hiPSC differentiation protocol that enables the consistent generation of full-thickness skin organoids containing hair follicles, sebaceous glands, and nerve fibers *in vitro* (Ahmed I. et al., 2024). This protocol specifically addresses the batch-to-batch variability issue prevalent in previous methods by standardizing the embryoid body formation step, thereby achieving high-fidelity simulation of skin appendages. Brightfield imaging confirmed the progressive development of hair follicles in these organoids by Day 65 of culture (Figure 4a). Immunofluorescence analysis further validated the structural complexity of the organoids, demonstrating proper epidermal stratification with KRT14^+^ basal and KRT10^+^ suprabasal layers, SOX2^+^ dermal papilla cells localized adjacent to the basal layer, and TUJ1^+^ neurites confirming neural components (Figure 4b). Furthermore, their subsequent work demonstrated the remarkable complexity of these organoids, revealing that they can develop into structures containing eccrine sweat glands in addition to hair follicles and sebaceous glands (Ahmed I. A. et al., 2024). Collectively, these studies establish a comprehensive platform for generating physiologically relevant skin organoids with progressively characterized complexity, laying a solid foundation for future psoriasis disease modeling and drug screening applications. Nevertheless, although this protocol has demonstrated the feasibility of replicating complex skin structures *in vitro*, it has not yet been directly applied to the simulation of the pathological mechanisms of psoriasis. In contrast, organoid systems derived from murine cells have been directly employed for psoriasis modeling. Jiang et al. constructed a mouse skin organoid system by isolating primary dermal and epidermal cells from neonatal BALB/c mice and co-culturing them in Transwell inserts at a 9:1 ratio. In this 3D culture system, the cells spontaneously self-organized into a planar skin-like structure comprising stratified epidermis and underlying dermis, which is a characteristic feature of ASC-derived organoids. Using this organoid platform, they functionally validated the LGALS9-CD44-HMGB2 mechano-chemical signaling axis *in vitro*, demonstrating that dermal fibroblasts, upon receiving LGALS9 signals from dendritic cells, remodel the extracellular matrix and subsequently promote basal epidermal cell proliferation through HMGB2-RRM2 signaling (Jiang et al., 2024). Although derived from murine cells, this system represents a robust *in vitro* organoid model that enables controlled mechanistic dissection of psoriatic pathogenesis, complementing human PSC-derived platforms.

**FIGURE 4:**
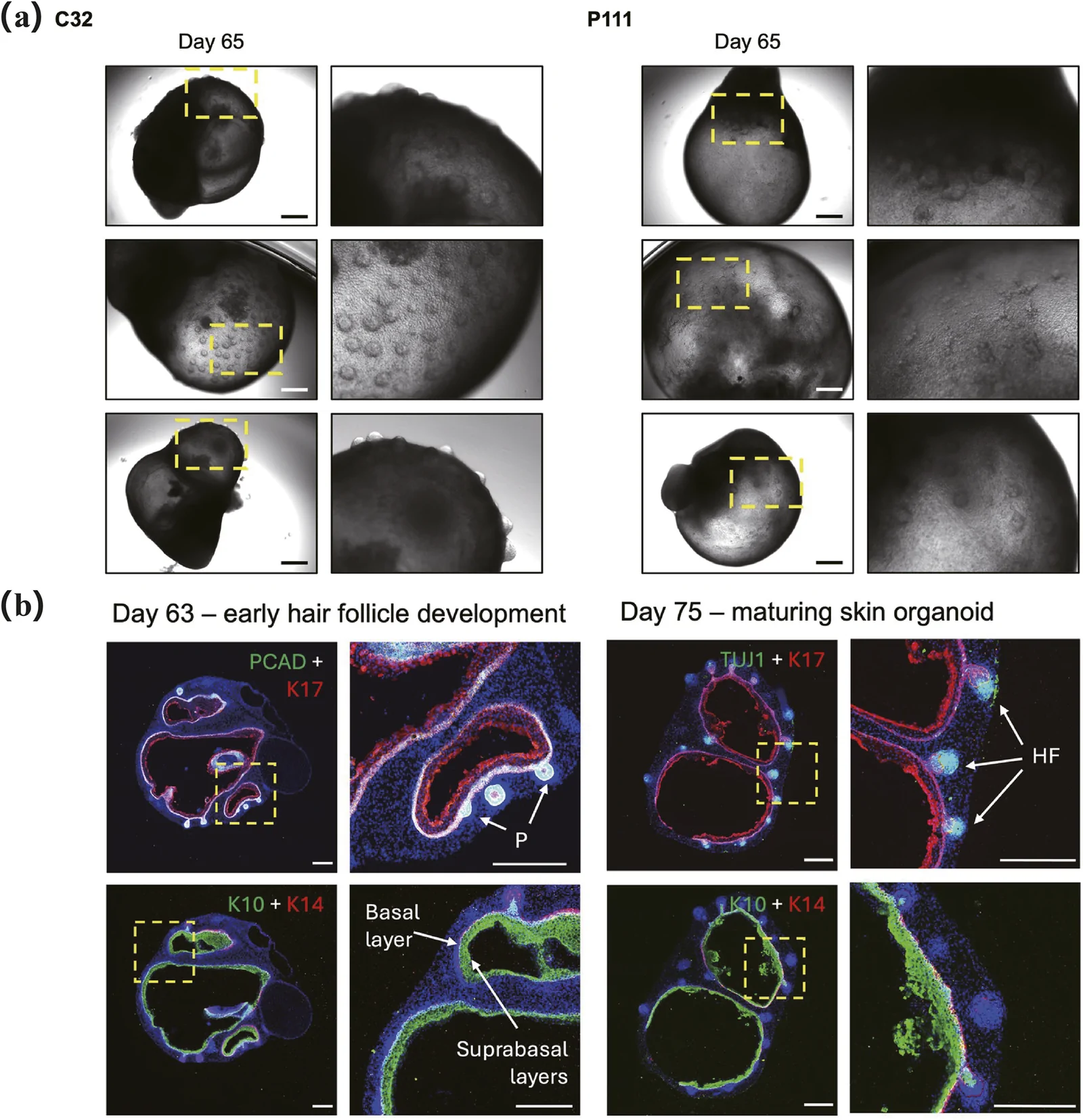
**(a)** Development of hair follicles in hiPSC-derived skin organoids. Brightfield images of representative SKOs at Day 65 of development, derived from P111 (CD34^+^ placental cell-derived) and C32 (skin fibroblast-derived) hiPSC lines. Early hair follicles are identifiable as circular cell clusters protruding from the organoid surface (dashed boxes indicate magnified regions). Scale bars: 500 µm. **(b)** Immunofluorescence characterization of mature skin organoids. Immunofluorescence analysis of SKOs at Day 75 of development. KRT14 (red) marks the epidermal basal layer, while KRT10 (white) labels the suprabasal differentiated layers. SOX2^+^ dermal papilla cells (green) are localized adjacent to the basal layer, recapitulating the hair follicle induction niche. TUJ1^+^ neurites (magenta) confirm the presence of neural components. DAPI (blue) stains cell nuclei. Dashed yellow boxes indicate magnified regions. HF, hair follicle; P, hair placode. Scale bar: 300 µm. Adapted from (Ahmed et al., 2024a), Copyright 2024a CC BY.

Building upon the structural foundation laid by Ahmed (Ahmed I. et al., 2024; Ahmed I. A. et al., 2024), and the inflammatory induction strategies validated in organoid systems (Jiang et al., 2024), we propose that the next frontier lies in integrating these two complementary approaches by generating hiPSC-derived, appendage-bearing human skin organoids and subsequently introducing inflammatory drivers such as cytokine cocktails or immune cell co-culture. This combined strategy would not only address the current lack of skin appendages in most models but also enable patient-specific *in vitro* recapitulation of the psoriatic inflammatory milieu, thereby offering a more faithful platform for mechanistic studies and drug screening.

#### 2.2.2 Psoriatic skin-on-a-chip

Beyond structural reconstruction, Skin-on-a-Chip (SoC) technology addresses the need to model the dynamic vascular microenvironment. Angiogenesis and the subsequent trans-endothelial migration of immune cells are hallmarks of the disease, processes that are heavily influenced by hemodynamic forces. To bridge this gap, Skin-on-a-Chip (SoC) technology has emerged as a transformative platform. By culturing skin tissues in dynamic, controlled microfluidic environments, SoC systems not only improve barrier function but also allow the integration of a perfusable vasculature (Mohammadi et al., 2016; Guan et al., 2017). This capability enables the recapitulation of the hemodynamic forces and continuous immune cell recruitment that drive disease progression, offering a level of pathophysiological fidelity unattainable in static cultures (Fernandez-Carro et al., 2022).

Shin et al. (Shin et al., 2020) developed an advanced 3D psoriatic skin model (pHSCs) that successfully integrated patient-derived T cells within a static Transwell culture system. Although this model lacked a perfusable vascular network, it represented a critical step toward immunocompetent disease modeling. As illustrated in Figure 5a, this model employed a schematic workflow to construct a human skin equivalent with integrated CD4^+^ T cells. Confocal imaging confirmed the successful infiltration of CD3^+^ T cells (green) into the dermal region beneath the K14^+^ epidermis (red), while quantitative analysis revealed that the presence of the epidermis markedly enhanced both the depth and extent of T cell migration. This model successfully reproduced the hallmark pathological process of T cell infiltration into the epidermis and mimicked the psoriatic inflammatory milieu, including epidermal hyperplasia. Its utility was further demonstrated by evaluating clinical therapeutics, such as anti-IL-17 antibodies, validating its potential for drug screening. However, despite these advances, the model relied on static Transwell culture systems lacking a perfusable vascular network (Zhou et al., 2024), thereby limiting its ability to fully simulate the hemodynamic forces and continuous immune cell recruitment observed *in vivo*. Thus, this model is best considered a critical precursor rather than a true SoC system.

**FIGURE 5:**
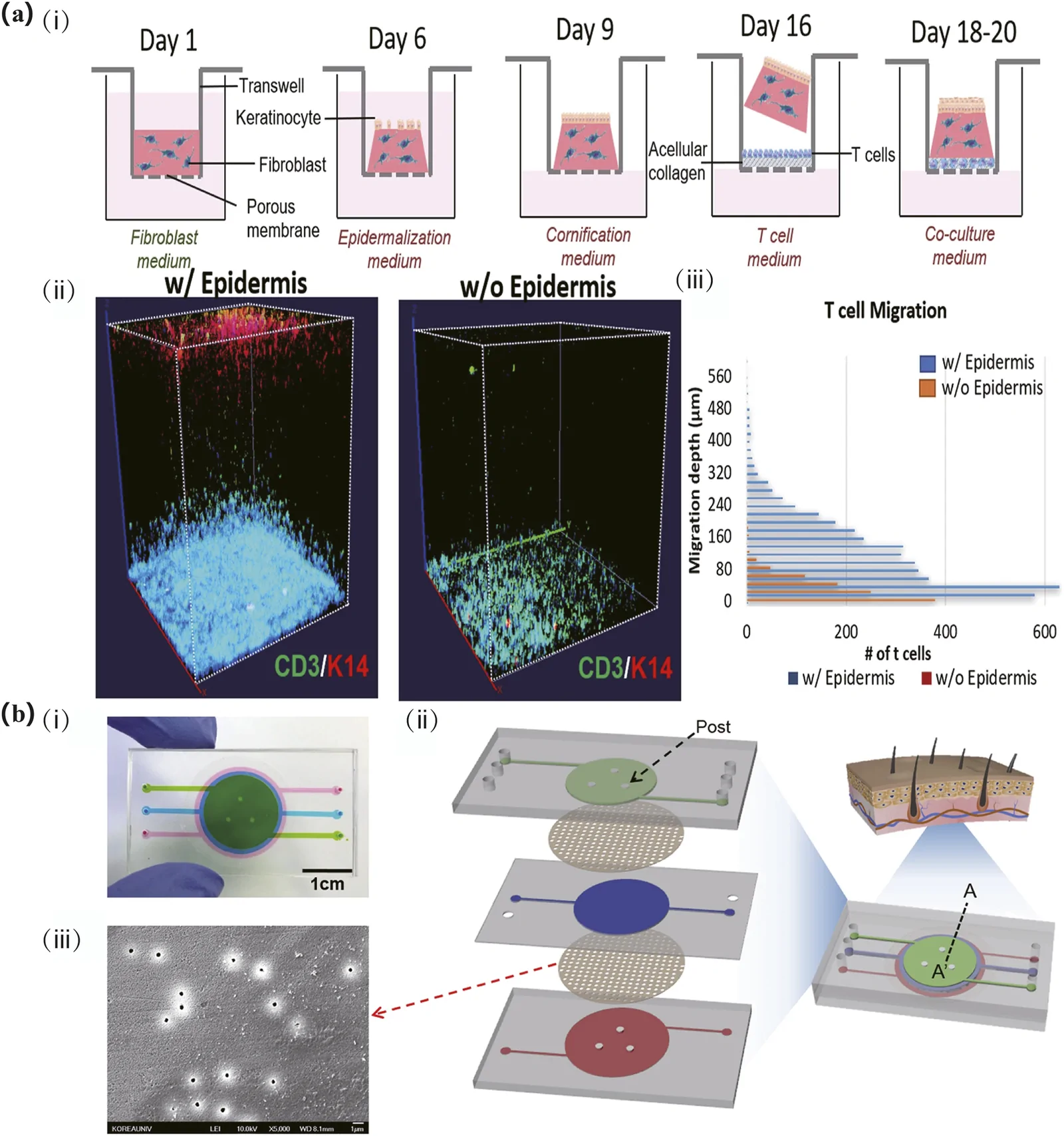
**(a)** Recapitulation of T cell infiltration in an immunocompetent 3D skin model. (i) Schematic workflow for constructing the human skin equivalent (HSC) with integrated CD4^+^ T cells. (ii) 3D confocal imaging confirms the infiltration of CD3^+^ T cells (green) into the dermal region of the HSC beneath the K14^+^ epidermis (red); nuclei are stained with DAPI (blue). (iii) Quantitative analysis comparing HSCs with and without the epidermis shows that the presence of the epidermis markedly enhances the depth and extent of T cell migration into the dermis. Adapted from (Shin et al., 2020), Copyright 2020 CC BY 4.0. **(b)** Design and fabrication of the skin-on-a-chip device. **(i)** Photograph of the assembled device filled with three colored solutions for visualizing the three stacked cell culture chambers. (ii) 3D schematic of the skin-on-a-chip system, comprising three PDMS layers separated by two porous PET membranes. (iii) SEM image of the PET porous membrane. Adapted from (Wufuer et al., 2016), Copyright 2016 CC BY 4.0.

To bridge this gap between static tissue models and the dynamic *in vivo* environment, the integration of microfluidic technology has emerged as a logical next step. Ren et al. (Ren et al., 2021) introduced microfluidic technology to construct a vascularized 3D human skin-on-a-chip (SoC). Their micropillar-based platform enabled precise spatial control over multiple cell types within a 3D collagen matrix, establishing a tri-cellular architecture consisting of an epidermis (HaCaT keratinocytes), a dermis (3D collagen gel), and a perfusable vascular endothelium (HUVECs). This architecture formed a functional blood-skin barrier. Using this platform, the study quantitatively investigated the mechanisms of T cell migration, specifically verifying how psoriasis-associated chemokines drive trans-endothelial and trans-epithelial migration under flow conditions. Furthermore, the model successfully recapitulated the TNF-α-induced inflammatory cascade, offering a robust platform for dissecting the dynamic mechanisms of psoriatic inflammation.

An earlier effort to incorporate vascular components into a chip-based platform was reported by Wufuer et al. (Wufuer et al., 2016), who developed a three-layer skin-on-a-chip model comprising epidermal, dermal, and endothelial layers. As depicted in Figure 5b, the assembled device featured three stacked cell culture chambers visualized with colored solutions, with a 3D schematic illustrating the three PDMS layers separated by two porous PET membranes, and SEM imaging confirming the porous structure of the PET membrane. By perfusing TNF-α through the dermal layer, the authors successfully induced inflammation and edema, and demonstrated the model’s utility in evaluating the anti-inflammatory effects of dexamethasone. Although this study did not focus on the specific immunopathology of psoriasis, it was a pioneering work that proved the feasibility of using multi-layered, vascularized skin chips to model skin inflammation and test therapeutic interventions. This work laid the groundwork for subsequent psoriasis-specific SoC development.

Collectively, these studies illustrate a clear trajectory from static immunocompetent models to dynamic vascularized SoC platforms, with early chip-based inflammation models providing foundational proof of concept. However, despite this progress, current SoC systems still face several inherent limitations. While the integration of perfusable endothelium represents a major advancement, existing models often simplify the skin structure by excluding skin appendages, which are the key microenvironments for psoriasis inflammation. Moreover, most chips typically have immune compartments that are limited to specific T cell subsets and cannot cover the full picture of the innate and adaptive immune crosstalk observed in patients. Therefore, future iterations must strive to achieve a balance between microfluidic complexity and biological fidelity, incorporate multi-cellular immune microenvironments and standardized protocols, in order to enable long-term cultivation and high-throughput drug screening.

#### 2.2.3 3D bioprinted psoriasis models

While organoids and skin-on-a-chip have advanced our understanding of specific pathological features, these platforms do not inherently address the challenge of precise spatial organization. Traditional self-assembled skin models are fundamentally limited by insufficient control over cell distribution and poor reproducibility, preventing the accurate recapitulation of the spatial heterogeneity and complex microenvironments characteristic of psoriasis (Li and Coombs, 2018; Abellan Lopez et al., 2023). Consequently, 3D bioprinting has emerged as a transformative solution to these challenges, enabling the controlled spatial deposition of multiple cell types and biomaterials to construct highly reproducible, personalized pathological models (Van Rietbergen et al., 1999; Landers et al., 2002).

Compared with conventional manual casting and self-assembly approaches, 3D bioprinting offers distinct advantages in constructing psoriatic skin models. It enables precise spatial control over cell distribution and tissue architecture (Phang et al., 2022; Mallya et al., 2025), which is essential for recapitulating the complex dermal-epidermal interface and vascular network characteristic of psoriatic lesions. Moreover, the automated nature of bioprinting improves reproducibility and scalability, facilitating high-throughput drug screening applications. Although these advantages have been demonstrated, the application of 3D bioprinting specifically to psoriasis remains in its early stages, with currently published peer-reviewed original research still relatively limited, highlighting both the nascent state of the field and the urgent need for model development.

To date, the most well-characterized fully 3D-bioprinted psoriatic skin model was developed by Peng et al. (Peng et al., 2025), who constructed a vascularized, full-thickness psoriatic skin model via 3D bioprinting. By co-printing fibroblasts, keratinocytes, and vascular endothelial cells within a precisely defined grid-like architecture, they successfully recapitulated the pathological dermal vascular network, a key feature of psoriatic lesions. This approach established an intact vascular network beneath a stratified epidermis, with bioprinted constructs maintaining cell viability above 90% after 7 days in culture and achieving transepithelial electrical resistance (TEER) values of approximately 383 kΩ, reflecting near-native barrier integrity. Following air-liquid interface (ALI) culture and cytokine induction, the model exhibited hallmark psoriatic phenotypes, including significant epidermal hyperplasia and the upregulation of inflammatory cytokines alongside the downregulation of differentiation markers. Structural and functional analyses confirmed that the model closely mimics the pathological hallmarks of psoriasis. Pharmacological validation further confirmed the model’s predictive utility: treatment with tofacitinib or danshensu reduced IL-22 and TNF-α expression by over 60% and increased loricrin expression nearly 2-fold, demonstrating dose-dependent therapeutic responses consistent with clinical outcomes.

Nevertheless, this 3D-bioprinted psoriasis model still faces several translational bottlenecks. First of all, the long-term structural stability remains a concern; the degradation kinetics of bioinks are often unoptimized, leading to structural collapse during the extended culture periods required for chronic disease modeling. Second, the reliance on immortalized cell lines compromises physiological relevance. These cells often exhibit impaired differentiation plasticity and form a thinner stratum corneum compared to primary human keratinocytes, which may skew drug permeability and efficacy assessments. Third, the absence of integrated immune cells, particularly Th17 and Tc17 cells, which are central to psoriasis pathogenesis, limits the model’s capacity to fully recapitulate the chronic inflammatory milieu. While Peng et al. induced inflammation via exogenous cytokine stimulation, this approach fails to capture the dynamic T cell-keratinocyte crosstalk that drives lesion persistence and relapse *in vivo*. This absence of integrated immune components is not unique to psoriasis models but reflects a pervasive gap across most bioprinted skin models, as highlighted in a recent comprehensive review (Ulloa-Fernández et al., 2026).

Moving forward, the field must address these limitations to fully realize the potential of 3D bioprinting. Future research should focus on developing bioinks with tunable degradation rates to support long-term culture and incorporating primary patient-derived cells to better capture individual disease variability. Notably, technological strategies established in bioprinting for other inflammatory skin conditions offer valuable references for psoriasis model refinement. Liu et al. (Liu et al., 2020) demonstrated that incorporating vascular endothelial cells into a bioprinted atopic dermatitis model enhanced chronic inflammation and improved drug screening accuracy, a strategy that is directly transferable to psoriatic constructs. Nornberg et al. (Nörnberg et al., 2025) developed a bioprinted gelatin-dialdehyde starch (Gel/DAS) hydrogel patch for controlled topical drug delivery, suggesting that future psoriatic models could integrate therapeutic release platforms for *ex vivo* efficacy testing. Kim et al. (Kim et al., 2021) constructed a vascularized, perfusable full-thickness diabetic skin model incorporating a hypodermis layer, highlighting the importance of including subcutaneous adipose tissue to model systemic inflammatory influences on lesion development. Adapting these proven engineering strategies to the Th17-specific immune microenvironment of psoriasis represents a promising direction for future development. Ultimately, integrating bioprinting with multi-omics analysis will be crucial for transforming these complex *in vitro* models into robust platforms for precision dermatology.

## 3 Common challenges and future directions of existing models

To ensure that our evaluation is grounded in pathophysiology rather than engineering novelty, we conducted a horizontal comparison of various 3D psoriasis models across five dimensions selected to assess their fidelity in recapitulating the complex pathophysiology of psoriasis. These dimensions include structural complexity, which assesses the model’s ability to mimic the stratified epidermis and dermal-epidermal junction; immune integration strategy, evaluating the presence and interaction of key immune cells to simulate the core IL-23/Th17 inflammatory axis; vascular/neurogenic capability, examining the inclusion of components critical for modeling angiogenesis and the brain-skin axis; scalability, referring to the ease of standardized production and cost-effectiveness; recommended biological composition, specifying the core cellular components required for each model category, including keratinocytes, fibroblasts, immune cells, endothelial cells, and patient-derived cells, which serves as the fundamental determinant of what biological questions a model can address; and primary application, describing the specific research scenarios for which each model is most appropriate.

Among these, the “Self-Assembled (Patient-derived)” category refers specifically to models constructed by co-culturing primary keratinocytes and fibroblasts derived directly from patients, where fibroblasts are cultured to generate their own extracellular matrix, forming a scaffold-free dermal layer. This approach preserves patient-specific genetic and epigenetic characteristics, as well as the biochemical composition of the extracellular matrix, making it distinct from stem cell-derived organoids. It should be noted that skin organoids are excluded from this comparison. Unlike the other models, which are engineered constructs assembled from pre-differentiated cells and exogenous scaffolds, organoids are self-organizing entities derived from stem cells (Lee et al., 2020; Lei et al., 2023). Their primary value lies in recapitulating developmental processes and complex appendages (Lee et al., 2018), rather than primarily in scalability or standardized immune integration. Including them in a dimension-based engineering comparison would conflate distinct biological paradigms. Therefore, the following comparison focuses on four model categories that share a common engineering logic.

The detailed comparison based on these criteria is presented in Table 3. To assist researchers in navigating this information, we emphasize that biological composition, rather than fabrication method alone, determines model suitability. As noted in Table 3 legend, the same biological components can often be implemented across different fabrication platforms depending on experimental needs. Readers are encouraged to first clarify their research objective and required biological components, then identify the most appropriate platform based on the feature comparison in Table 3, while keeping in mind the trade-offs between biological complexity, experimental throughput, and technical feasibility.

**TABLE 3:** Comparative analysis of key features in 3D psoriasis models.

Model category	Structural complexity	Immune integration strategy	Vascular/Neural capability	Scalability	Recommended biological composition	Primary application scenario
Commercial Models	High standardization; often lacks patient-specific genetic background	Exogenous cytokine stimulation (IL-17/TNF-α); lacks cellular immune crosstalk	Static culture; no vasculature/innervation	High; standardized, mass-producible	Keratinocytes + Fibroblasts	High-throughput drug screening barrier function testing; toxicity assessment
Self-Assembled (Patient-derived)	Retains patient-specific ECM and genetic memory; long culture time	Co-culture with autologous immune cells; recapitulates IL-23/Th17 axis	Limited by static culture conditions	Moderate; labor-intensive, donor-dependent	Keratinocytes + Fibroblasts + Patient-derived cells + (Optional) T cells/DCs	Mechanism research; personalized medicine; target validation
Organ-on-Chip	Microfluidic control; can induce shear stress effects	Dynamic immune cell recruitment; Limited by simplified immune subsets	Perfusable endothelium; neural integration not yet achieved	Low; complex setup, specialized equipment	Keratinocytes + Fibroblasts + Endothelial cells + Microfluidic perfusion + (Optional) Immune cells	Dynamic physiological simulation; vascular-immune crosstalk; immune cell transmigration
3D Bioprinting	Precise spatial control; can engineer vascular networks	Compatible with multi-cellular bioinks; technical complexity high	Vascularization achieved in some constructs; neural integration lacking	Moderate-High; automated potential, bioink limitations	Flexible: Keratinocytes + Fibroblasts ± Immune cells ± Endothelial cells ± Patient-derived cells	Complex tissue architecture modeling; precision spatial control; customized disease modeling

The Primary Application Scenario column reflects common use cases, not rigid assignments. This table is intended as a practical reference for model selection, not a restrictive classification. For guidance on how to select a platform based on your research objective and required biological components, please refer to the main text.

Although the existing 3D skin models have made significant progress in reproducing the typical pathological features of psoriasis, we still face a substantial gap between simple structural mimicry and the reconstruction of complex physiological functions. In fact, the six evaluation dimensions outlined above converge on three core limitations that pervade current *in vitro* models, including vascular insufficiency, neural absence, and immune dynamics, which we discuss in detail below.

### 3.1 Lack of physiological microenvironment crosstalk

Most current models are limited to simple co-cultures of the epidermis and dermis, often neglecting the crucial processes of vascularization and nerve innervation in the pathogenesis of psoriasis.

Psoriasis is not merely an epidermal disease; angiogenesis is a key event in its early stage (Chua et al., 2009). Existing static models lack perfusion-type vascular networks (Mirzapour-Shafiyi et al., 2025), making it impossible to simulate the process of immune cells extravasating from the blood to the tissue, and also difficult to evaluate the efficacy of anti-angiogenic drugs.

Moreover, psychological stress can lead to the emergence and recurrence of psoriasis (Lei et al., 2025), a process in which the nervous and immune systems are closely interconnected (Zhang et al., 2023). This connection is mediated by the brain-skin axis, where stress activates the hypothalamic-pituitary-adrenal (HPA) axis (A.R. Brunoni et al., 2014) and the sympathetic nervous system. Specifically, stress triggers the release of neuropeptides, such as Substance P (SP) and Calcitonin Gene-Related Peptide (CGRP), from cutaneous sensory nerve endings (Wijayanti et al., 2022). These neuropeptides bind to receptors on keratinocytes and immune cells, promoting the release of pro-inflammatory cytokines such as IL-1β, IL-6, and TNF-α (Marek-Jozefowicz et al., 2023). This process can further influence the maturation and expansion of Th17 cells, a key pathological pathway in psoriasis (Zheng et al., 2007; Ma et al., 2008).

However, most existing *in vitro* models remain largely devoid of functional nerve innervation (Zhu et al., 2016; Zhang and He, 2020), creating a significant gap in simulating this pathophysiological process. The absence of a neuro-immune interface in current constructs cuts off the communication between neuropeptides and immune cells, limiting our understanding of stress-induced recurrence. To address this, future disease modeling should aim to integrate sensory neurons or mimic neuro-immune crosstalk to better recapitulate the complex microenvironment of psoriatic skin under stress conditions.

In particular, stress responses vary considerably among individuals, from differences in neuropeptide release to variations in immune cell sensitivity. This may help explain why some psoriasis patients experience stress-induced flares while others do not. Generic models derived from healthy donors cannot capture this variability, reinforcing the argument for patient-specific platforms that preserve individual genetic and neuro-immunological backgrounds. In practical terms, skin organoids that already contain nerve fibers could serve as a starting point for modeling neuro-immune interactions (Ahmed I. et al., 2024), though how to integrate psoriatic inflammatory drivers into such systems remains an open question.

### 3.2 Challenges in simulating dynamic immune evolution

Psoriasis is a dynamic disease driven by the immune system (Woo et al., 2017), but current *in vitro* models mostly appear as static snapshots. Conventional cytokine-induced models often rely on the exogenous addition of inflammatory factors to the culture medium, which fails to recapitulate the continuous recruitment, activation, and expansion of immune cells guided by physiological chemokine gradients.

More fundamentally, the inability to model the temporal progression of the disease, from a healthy state through external stimuli to immune outbreak and eventual resolution or relapse, remains a critical bottleneck. Most models are evaluated at a single endpoint, providing only a snapshot of the inflammatory state rather than capturing the dynamic evolution of the disease. This limitation makes it difficult to study the initiation mechanisms of psoriasis or to assess the time-dependent efficacy of therapeutic interventions.

Furthermore, while some advanced co-culture systems integrate pre-activated immune cells, the majority of existing platforms lack a perfusable vascular network. This restricts the simulation of continuous immune cell infiltration and the dynamic crosstalk between circulating immune cells and resident skin cells that drives lesion progression and relapse.

### 3.3 Translational gap between standardization and personalization

At the translational medicine level, we encounter a contradiction where reproducibility and specificity are often difficult to achieve simultaneously. Commercialized skin models are usually derived from healthy donors, masking the unique epigenetic memory and genetic background of psoriasis patients (Rohrbeck et al., 2024). Although iPSC technology offers the possibility of constructing patient-specific models (Wiegand and Banerjee, 2019), its low differentiation efficiency and significant batch-to-batch variations make it difficult to meet the strict requirements of high-throughput drug screening for standardization (Doss and Sachinidis, 2019; Kim et al., 2024).

However, the future of *in vitro* modeling lies in stratifying applications to balance this trade-off. While standardized models will remain the cornerstone for high-throughput toxicity and efficacy screening, patient-specific models are indispensable for “precision dermatology.” These personalized platforms are crucial for dissecting pathogenesis in non-responders to biologic therapies and for screening drugs tailored to individual genetic backgrounds (Mastorino et al., 2022). Therefore, the next-generation of models aims to leverage iPSC technology to offer scalable platforms that can also be customized when clinical complexity demands it.

## 4 Conclusion

As the global burden of psoriasis continues to increase, the construction of precise *in vitro* pathological models holds crucial clinical value for breaking through existing treatment bottlenecks. In recent years, *in vitro* model technology has undergone an iterative transition from structural mimicry to functional mimicry, evolving from traditional 3D models based on cytokine induction or immune-cell co-culture to biomimetic skin systems integrating microfluidics and bioprinting. Although these two approaches have demonstrated complementary advantages in drug screening and mechanism analysis, the current models have not yet fully bridged the gap between *in vitro* simulation to clinical reality: complex immune-inflammatory circuits remain incompletely recapitulated, the absence of skin appendages such as hair follicles and sweat glands distorted the tissue microenvironments, and the lack of patient-specific genetic background limits both long-term culture stability and pathological fidelity.

Moving forward, the core of the next-generation *in vitro* models lies in transcending single-dimensional limitations and evolving towards high fidelity, long-term culture and individualization. Although current models still lack certain physiological structures such as vasculature and skin appendages, prioritizing individualization is fundamentally justified. Psoriasis is a highly heterogeneous disease driven by complex genetic susceptibility and patient-specific immune memory. Without incorporating this patient-specific background, even structurally complex models derived from healthy donors essentially simulate healthy skin under inflammatory stress rather than true psoriatic pathology, rendering them inherently limited for studying disease recurrence and personalized drug responses. Therefore, achieving individualization is not only an ethical imperative for implementing the 3R principles and reducing reliance on animal experiments, but also the inevitable pathway toward advancing psoriasis treatment from symptomatic management to precision medicine. Building an *in vitro* platform that can dynamically recapitulate the full disease spectrum will provide more translational evidence for dissecting complex pathogenesis and developing individualized therapies.
